# Human Calmodulin Mutations

**DOI:** 10.3389/fnmol.2018.00396

**Published:** 2018-11-13

**Authors:** Helene H. Jensen, Malene Brohus, Mette Nyegaard, Michael T. Overgaard

**Affiliations:** ^1^Section for Biotechnology, Department of Chemistry and Bioscience, Aalborg University, Aalborg, Denmark; ^2^Department of Biomedicine, Aarhus University, Aarhus, Denmark

**Keywords:** calmodulin, cardiac arrhythmia, calmodulinopathy, CPVT, LQTS, *CALM1*, *CALM2*, *CALM3*

## Abstract

Fluxes of calcium (Ca^2+^) across cell membranes enable fast cellular responses. Calmodulin (CaM) senses local changes in Ca^2+^ concentration and relays the information to numerous interaction partners. The critical role of accurate Ca^2+^ signaling on cellular function is underscored by the fact that there are three independent CaM genes (*CALM1-3*) in the human genome. All three genes are functional and encode the exact same CaM protein. Moreover, CaM has a completely conserved amino acid sequence across all vertebrates. Given this degree of conservation, it was long thought that mutations in CaM were incompatible with life. It was therefore a big surprise when the first CaM mutations in humans were identified six years ago. Today, more than a dozen human CaM missense mutations have been described, all found in patients with severe cardiac arrhythmias. Biochemical studies have demonstrated differential effects on Ca^2+^ binding affinities for these CaM variants. Moreover, CaM regulation of central cardiac ion channels is impaired, including the voltage-gated Ca^2+^ channel, Ca_V_1.2, and the sarcoplasmic reticulum Ca^2+^ release channel, ryanodine receptor isoform 2, RyR2. Currently, no non-cardiac phenotypes have been described for CaM variant carriers. However, sequencing of large human cohorts reveals a cumulative frequency of additional rare CaM mutations that raise the possibility of CaM variants not exclusively causing severe cardiac arrhythmias. Here, we provide an overview of the identified CaM variants and their known consequences for target regulation and cardiac disease phenotype. We discuss experimental data, patient genotypes and phenotypes as well as which questions remain open to understand this complexity.

## Introduction

For generations, a large Swedish family presented with repeated episodes of syncope and cardiac arrest in response to exercise or emotional stress. Several family members were diagnosed with the inherited disorder catecholaminergic polymorphic ventricular tachycardia (CPVT), which is often fatal due to a high risk of ventricular fibrillation and sudden cardiac death (SCD). Indeed, two of the 13 affected individuals died from SCD, both at a young age. In 2012, we linked the disease to a mutation in the gene *CALM1*, which encodes the calcium (Ca^2+^) sensor calmodulin (CaM) (Nyegaard et al., 2012). The identification of a human CaM missense mutation came as a dramatic surprise to the CaM research field; CaM is exceptionally conserved across species with all vertebrate *CALM* genes encoding identical proteins, and human mutations had not previously been reported. The slow evolution of CaM emphasizes the strong selection pressure against even minor changes in the protein sequence (Halling et al., 2016). Further, CaM regulates more than 300 intracellular targets, each interaction with unique facets of binding sites, Ca^2+^-dependency, target affinity, and functionality (Shen et al., 2005; O’Connell et al., 2010). With this versatility in mind, it was believed that mutations in CaM could not be tolerated.

After our initial finding, a number of CaM mutations have been identified in patients with severe cardiac arrhythmia disorders involving recurrent syncope, ventricular fibrillation, and in some instances SCD under adrenergic stimulation (Table 1). The vast majority of these mutations are *de novo* and carriers present with disease phenotypes early or very early in childhood, in some cases even before birth. In addition to CPVT, carriers suffer from long QT syndrome (LQTS), and one individual was diagnosed with idiopathic ventricular fibrillation (IVF). The link between CaM mutations and these arrhythmias has primarily been attributed to impaired regulation of the cardiac ryanodine receptor isoform 2 (RyR2), and the cardiac L-type voltage gated Ca^2+^ channel isoform 1.2 (Ca_V_1.2) (Table 1).

**Table 1:** Pathogenic CaM mutations affect Ca^2+^ and target binding as well as target regulation.

CaM mutation		Patients		CaM protein effect			Heart beat effect			Effect on Ca_V_1.2		Effect on RyR2				
						
HGVS nomen-clature	Mature CaM	Gene	Diagnosis	EF-hand (coordinates Ca^2+^)	K_D_ C-lobe–fold change	K_D_ N-lobe-fold change	Action potential duration	Ca^2+^ Transient entrainment	Zebrafish heart rate	Binding to CI region	Ca^2+^ dependent inactivation (CDI)	CaM binding to RyR2		Channel inhibition		
												Low [Ca^2+^]	High [Ca^2+^]	Low [Ca^2+^]	Intermediate [Ca^2+^]	High [Ca^2+^]
				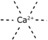	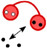	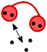	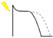	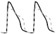	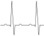	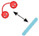	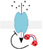	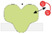	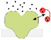	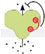	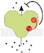	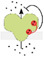

N54I	N53I	*CALM1*	CPVT^a^	−	0.9^l^/—^p,q^	1.2^l^/–^p,q^	—^s^	Strongly perturbed^r^	↑**^∗^**^l^		—^g,s^	↑^p,q^	— ^q^/**↑**^p^	**↓**^g,m,q^**/—**^p^	**↓**^p,q^	**↓**^p^**/—**^n^

F90L	F89L	*CALM1*	IVF^b^	−	8.3^o^	1.8^o^						**↓**^o^	**↓**^o^	**↓**^o^	**↓**^o^	**↓**^o^

D96H	D95H	*CALM3*	LQTS^c^	+												

D96V	D95V	*CALM2*	LQTS^d^	+	11.8—13.6^d,m,p^	0.3^m^/—^d,p^	**↑**^g,s^	Perturbed^r^		—^s^	**↓**^g,r^**/↓↓**^s^	↓^n^/—^q^/↑^p^	↓^n^ /↑^p,q^	**↓**^m^/—^p,q^	—^q^/**↓**^p^	**↓**^p^**/—**^n^

N98I	N97I	*CALM2*	LQTS^e^	+	8.3^e^	—^e^										

		*CALM1*	CPVT^a^													
		*CALM2*	LQTS^e^													
N98S	N97S	*CALM2*	LQTS^f^	+	3.3—4.0^l,p,q^	—^l,p,q^	**↑**^t^		↑**^∗^**^l^		**↓**^s,t^**/**—^g^	—^n,p^**/↑**^q^	—^n,p,q^	**↓**^g,m,q^**/—**^p,q^	**↓**^q^**/—**^p^	—^n,p^
		*CALM2*	CPVT + LQTS^f^													

A103V	A102V	*CALM3*	CPVT^g^	−	2.9^g^	0.9^g^	—^g^				—^g^	—^g^	—^g^	**↓**^g^		

E105A	E104A	*CALM1*	LQTS^h^	+												

		*CALM1*	LQTS^d^													
		*CALM1*	LQTS^d^													
D130G	D129G	*CALM1*	LQTS^i^	+	33.6—53.6^d,m,p^	0.8^m^/—^d,p^	**↑**^s,u^	Perturbed^r^	**↓**^w^	—^s^	**↓**^r,u^**/↓↓**^s^	**↓**^p,q^	**↓**^p,q^	**↓**^m^/—^p^/**↑**^q^	**↓**^p^	**↓**^p^**/—**^n^
		*CALM3*	LQTS^j^													

D130V	D129V	*CALM2*	LQTS^i^	+												

D132E	D131E	*CALM2*	CPVT + LQTS^e^	+	22.9^e^	—^e^										

D132H	D131H	*CALM2*	LQTS^k^	+	77.0^k^	**↓**^k^					**↓**^k^					

D132V	D131V	*CALM1*	LQTS^k^	+	63.5^k^	**↓**^k^					**↓**^k^					

D134H	D133H	*CALM2*	LQTS^e^	+	12.9^e^	—^e^										

Q136P	Q135P	*CALM2*	CPVT + LQTS^e^	+	6.5^e^	—^e^										

E141G	E140G	*CALM1*	LQTS^i^	+	10.8^i^	0.6^i^					**↓**^i^			**—**^i^		

		*CALM1*	LQTS^d^													
		*CALM1*	LQTS^i^													
F142L	F141L	*CALM1*	LQTS^i^	−	4.8–6.0^d,n,p^	—^d,p^	**↑**^s,v^	Weakly perturbed^r^		**↑**^s^	**↓**^r,v^**/↓↓**^s^	**↓**^q^**/—**^p^**/↑**^n^	—^n,p,q^	**↓**^n^**/**—^p^ **/↑**^q^	**↓**^p^	—^p^ **/↑**^n^
		*CALM3*	LQTS^c^													

*All published CaM variants are indicated with the associated phenotype and published experimental data in each independent case. All parameters are illustrated with small schematics. For all biochemical and cellular experiments, changes are given by values or arrows indicating increases/decreases relative to CaM-WT. Vertical lines indicate no difference. Lowered Ca^2+^-binding affinity of CaM mutants are given as fold increase of dissociation constants (K_D_ values) relative to CaM-WT. Data on CaM binding to RyR2 is pooled from immunoprecipitation, FRET, and CaM binding domain binding experiments. Data on RyR2 inhibition was pooled from experiments studying store-overload induced Ca^2+^ release (SOICR), Ca^2+^ spark/wave frequency in intact cells, lipid bilayer currents, and ryanodine binding. RyR2 experiments are pooled in low (0–150 nM), intermediate (1 μM), or high (10–200 μM) free Ca^2+^ concentrations. ^∗^Under β-adrenergic stimulation. Ca_V_1.2, voltage-gated Ca^2+^ channel isoform 1.2; HGVS, human genome variation society; RyR2, ryanodine receptor isoform 2. ^a^Nyegaard et al. (2012), ^b^Marsman et al. (2014), ^c^Chaix et al. (2016), ^d^Crotti et al. (2013), ^e^Makita et al. (2014), ^f^Jiménez-Jáimez et al. (2016), ^g^Gomez-Hurtado et al. (2016), ^h^Takahashi et al. (2017), ^i^Boczek et al. (2016), ^j^Reed et al. (2015), ^k^Pipilas et al. (2016), ^l^Søndergaard et al. (2015a), ^m^Søndergaard et al. (2015b), ^n^Søndergaard et al. (2017), ^o^Nomikos et al. (2014), ^p^Vassilakopoulou et al. (2015),^q^Hwang et al. (2014), ^r^Yin et al. (2014), ^s^Limpitikul et al. (2014), ^t^Yamamoto et al. (2017), ^u^Limpitikul et al. (2017), ^v^Rocchetti et al. (2017), ^w^Berchtold et al. (2016).*

Since CaM is encoded by three active genes and expressed in all cells, the CaM field is faced with intriguing questions and paradoxes at the genetic and phenotypic level. First, how can a single mutation in one of six CaM-encoding alleles dominantly cause SCD? Second, how can identical missense mutations cause LQTS in one patient and CPVT in another? Third, with an increasing number of new rare CaM missense mutations identified in sequencing databases of large human cohorts, could there be other phenotypes associated with CaM mutations? Improved understanding of the functional impact of CaM mutations may enable predictions of the genotype–phenotype relationship for variants in any of the three *CALM* genes.

In this review, we summarize and discuss the current knowledge on CaM mutations and their impact on the regulation of Ca_V_1.2 and RyR2, and address the few studies that suggest an involvement of other targets. Finally, we discuss the special genetic context of CaM and the implications for future studies.

## CaM, the Cellular Ca^2+^ Sensing Protein

Fast and compound changes in cytosolic Ca^2+^ concentration is the foundation for a wide number of cellular responses, including muscle contraction and neuronal firing (Clapham, 2007). Thus, at rest, the cytosolic Ca^2+^ concentration is maintained at ∼100 nM, but can rapidly increase to more than 100 μM, when Ca^2+^ channels open in the plasma membrane or in internal stores such as the sarcoplasmic reticulum (SR). Detection of this steep change in Ca^2+^ concentration depends on Ca^2+^ binding proteins. CaM is one of the major Ca^2+^ sensors that relay information on Ca^2+^ concentration to functionally modulate target proteins (known as calmodulation). CaM is synthesized as a 149 amino acid protein, however, the initiator Met residue is removed upon translation, leaving 148 amino acid residues in the mature protein (Sasagawa et al., 1982). This has led to some confusion in the numbering of CaM variant positions. The Human Genome Variant Sequence (HGVS) nomenclature (den Dunnen et al., 2016) recommends to count the initiator Met as residue number 1, while the CaM protein community tends to leave the residue out as it has no functional role in the mature protein. Throughout this paper we will use the HGVS nomenclature.

**FIGURE 1:**
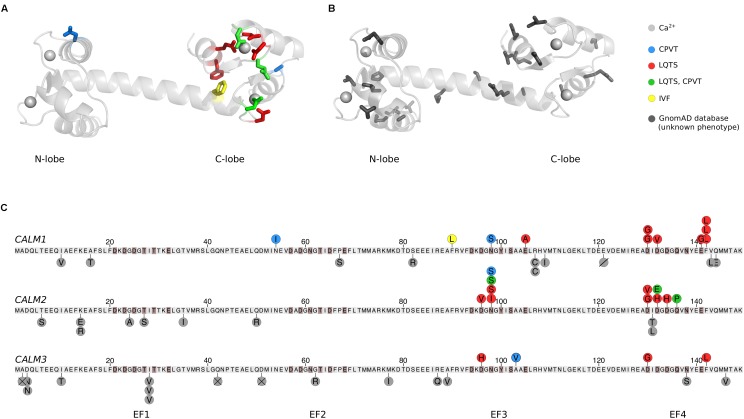
Human calmodulin mutations. **(A)** The calmodulin (CaM) protein has two lobes connected by a flexible α-helical linker. Each lobe has two EF-hands, which each can coordinate a Ca^2+^ ion (gray spheres), generating four Ca^2+^ binding sites. Residues highlighted in color and stick representation are mutated in cardiac arrhythmia patients, where they are associated with CPVT, LQTS, IVF, or a combination of CPVT and LQTS as indicated. The structure was visualized in PyMOL (PDB: 1CLL) (Chattopadhyaya et al., 1992). **(B)** CaM residues shown in dark gray stick representation are found mutated in the GnomAD database. GnomAD includes 277,364 alleles from 138,632 individuals (Lek et al., 2016). **(C)** Overview of mutations in each of the three human *CALM* genes, displayed as the translated protein with the initiator-Met as residue 1. Residues highlighted in brown participate in Ca^2+^ coordination. Published arrhythmogenic human variants are indicated by circles above the sequences, with colors indicating the disease phenotype. Dark gray circles below the sequence are individual allele variations observed in GnomeAD without a known phenotype. Amino acid variants are shown by letters, a slash (deletion), or a cross (frame-shift or premature stop codon). CPVT, catecholaminergic polymorphic ventricular tachycardia; LQTS, long QT syndrome; IVF, idiopathic ventricular fibrillation.

The structure of CaM reflects its refined Ca^2+^ sensing abilities (Kretsinger et al., 1986; Chattopadhyaya et al., 1992). CaM is a 16.7 kDa protein consisting of two lobes connected by a flexible and unstructured or α-helical linker (Figures 1A,B). Each lobe has two EF-hands, which can each coordinate one Ca^2+^ ion (Figures 1A,B, gray spheres). The C-terminal lobe of CaM binds Ca^2+^ with six times higher affinity (*K*_D_ 2.5 μM) than the N-terminal lobe (*K*_D_ 16 μM), allowing CaM to sense Ca^2+^ across a wide concentration range (Linse et al., 1991; Søndergaard et al., 2015a). Hydrophobic patches on the inside of each lobe recognize binding motifs on interaction partners, and thereby facilitate CaM binding and target regulation (Tidow and Nissen, 2013). Ca^2+^ binding to CaM and CaM binding to target proteins allosterically affect the affinity of each other, and targets specifically modulate the conformation of CaM. In this way, the small CaM protein displays a plethora of binding and regulation properties.

## CaM Conservation and Mutations

Despite the remarkable conservation of CaM, 26 cases of arrhythmogenic mutations have now been identified in humans. Their positions in CaM are indicated on a CaM structure in Figure 1A and on the CaM sequence in Figure 1C. Strikingly, the mutations are primarily found in the C-terminal lobe and most affect residues involved in Ca^2+^ coordination, dramatically reducing Ca^2+^ affinity (Table 1). One interesting exemption is the mutation initially identified in the large Swedish family, CaM-N54I. This mutation is unique since it (1) resides in the N-terminal lobe and (2) neither coordinates Ca^2+^ nor is part of the hydrophobic target binding patches. Biochemical and cellular experiments have been employed to model and explain how CaM mutations lead to arrhythmic phenotypes. The results from these studies are discussed in the following.

### CaM Mutations Disturb Heart Rhythm

The composite effect of CaM mutations on heart function has been investigated using different experimental model systems. In zebrafish, the CaM-N54I and -N98S mutations caused increased heart rate upon β-adrenergic stimulation, which is in line with the CPVT phenotype observed for patients with these mutations (Søndergaard et al., 2015a). Similarly, the LQTS-mutation D130G increased zebrafish heart rate (Berchtold et al., 2016). In cultures of ventricular cardiomyocytes, expression of LQTS-associated CaM mutations leads to prolonged action potentials (APs), in some cases spilling over to the next stimulation and causing alternans (Limpitikul et al., 2014, 2017; Gomez-Hurtado et al., 2016; Yamamoto et al., 2017). Moreover, CaM-D96V was associated with early after-depolarizations (Gomez-Hurtado et al., 2016). Imaging of Ca^2+^ fluxes during electrical pacing of cardiomyocytes demonstrated dysregulated Ca^2+^ concentration in cells expressing LQTS-associated CaM-D96V, -D130G, -F142L, and in particular the CPVT-mutation N54I showed Ca^2+^ overload, Ca^2+^ reuptake errors, or alternans (Limpitikul et al., 2014, 2017; Yin et al., 2014).

Ca_V_1.2 and RyR2 are the two major Ca^2+^ channels involved in Ca^2+^ handling in the heart. Both channels are essential for stimulating – and importantly, terminating – heart contraction. Briefly, APs stimulate the opening of Ca_V_1.2 in the sarcolemma. This allows Ca^2+^ to enter the cell. The resulting increase in cytosolic Ca^2+^ stimulates opening of RyR2, thereby releasing large amounts of Ca^2+^ from the SR which ultimately leads to heart contraction (Sorensen et al., 2013). CaM binds to both Ca_V_1.2 and RyR2 and is important for the precise and timely gating of the channels in response to changes in Ca^2+^ concentration. Generally, CPVT is an SR Ca^2+^ handling disease, most often caused by RyR2 mutations, whereas LQTS involves dysregulation of ion-fluxes across the sarcolemma, e.g., Ca^2+^ flux disturbances caused by mutations in Ca_V_1.2 (Landstrom et al., 2017).

### Impaired Regulation of CaV1.2

CaM is tethered to the intracellular C-terminal tail of Ca_V_1.2 and functions as a Ca^2+^ sensor to stimulate channel closure when Ca^2+^ has entered the cell, a process termed calcium-dependent inactivation (CDI) (Brehm and Eckert, 1978; Peterson et al., 1999). Reduction of CDI was observed for several LQTS-causing CaM mutations, including D132H, D132V, and E141G (Boczek et al., 2016; Pipilas et al., 2016), whereas CDI was completely absent for LQTS-associated CaM mutations D96V, D130G, and F142L in HEK293 cells and adult guinea pig cardiomyocytes (Limpitikul et al., 2014). The CaM-N98S mutation is special in the sense that carriers display either CPVT or LQTS arrhythmias or both. Expression of this mutant slightly reduces CDI of Ca_V_1.2, whereas the strictly CPVT-causing mutation CaM-N54I showed no effect (Limpitikul et al., 2014; Yamamoto et al., 2017).

### Effects of CaM Mutations on RyR2

RyR2 opening is normally stimulated by the increase in Ca^2+^ upon Ca_V_1.2 opening. In response to the dramatic increase in cytoplasmic Ca^2+^ concentration (and the drop in SR luminal Ca^2+^ concentration), RyR2 closes. CaM acts as a gatekeeper, by modulating the open state probability of RyR2 in response to changes in the Ca^2+^ concentration (Fabiato, 1985; Xu and Meissner, 2004). Here, we have compiled the diverse experimental approaches used to evaluate whether CaM mutations affect RyR2 regulation under different Ca^2+^ concentrations (Table 1). The results are mixed and in some cases contradictory. The CPVT-associated CaM mutations, N54I, N98S, and A103V, all showed an increased level of RyR2 opening, that is, decreased inhibition by CaM (Hwang et al., 2014; Søndergaard et al., 2015b; Gomez-Hurtado et al., 2016). These results suggest that the molecular disease mechanism for the CPVT-causing CaM mutations is dysregulation of cardiac SR Ca^2+^ release, in line with CPVT-causing mutations in RyR2.

Surprisingly, LQTS-associated CaM mutations also caused differences in RyR2 binding and regulation (Hwang et al., 2014; Søndergaard et al., 2015b; Vassilakopoulou et al., 2015). However, in particular at low Ca^2+^ concentrations, the data are somewhat contradictory (Table 1). Curiously, CaM-F142L showed an increased binding affinity toward RyR2 in the apo-form, as well as an increased inhibitory effect on RyR2 in some assays, an effect opposite of all other CaM mutants analyzed (Søndergaard et al., 2017).

### Other Suggested and Potential Targets

Although mutations in Ca_V_1.2 can lead to LQTS, most cases of LQTS can be attributed to mutations in genes encoding the voltage-gated K^+^ channels K_V_7.1 (*KCNQ1*) and K_V_11.1 (*KCNH2*), as well as the voltage-gated Na^+^ channel Na_V_1.5 (*SCN5A*) (Modell and Lehmann, 2006). Although regulated by CaM, no clear effects of CaM mutations have been observed on Na_V_1.5 (Yin et al., 2014; Boczek et al., 2016; Rocchetti et al., 2017). K_V_7.1 utilizes CaM as a sensor of Ca^2+^ to stimulate opening (Ghosh et al., 2006). Whereas CaM-F142L did not show any effect on K_V_7.1 current (*I*_Ks_) (Rocchetti et al., 2017), CaM-N98S significantly shifted the half-activation of K_V_7.1 (Sun and MacKinnon, 2017). Further, the small-conductance Ca^2+^-activated K^+^ (SK) channel was decreased by several CaM mutations (Yu et al., 2016). Although SK channels play a minor role in ventricular myocytes, they are expressed in atrial myocytes, and interestingly, widely expressed in the nervous system where they play a major role in synaptic transmission (Adelman et al., 2012).

Ca^2+^/CaM-dependent kinase II (CaMKII) regulates a wide number of pathways and protein targets, but no significant effects were observed on CaMKII with either of the CaM variants N54I, D96V, N98S, D130G, or F142L (Hwang et al., 2014; Berchtold et al., 2016).

Although current literature suggests that Ca^2+^ channels are the main targets affected by CaM mutations, we hypothesize that other ion channels and potentially other signaling proteins may also be dysregulated if tested in greater detail. But how can one predict which targets are likely affected by CaM mutations? We believe that the Ca^2+^-dependency of target binding plays an important role. That is, proteins that bind to both the apo- and the Ca^2+^-form of CaM may be more sensitive to mutations in CaM than targets that only bind the Ca^2+^-form. Thus, CaM mutations may exert a dominant effect in cases where CaM remains associated with its target when the cell is at rest.

## Implications of the Genetic Architecture of *CALM* Genes

In humans, CaM is encoded by three different and independent loci; on chromosome 2 (*CALM2*), 14 (*CALM1*), and 19 (*CALM3*). Although there are differences in the genomic sequence, the three different transcripts are translated into the exact same protein (Fischer et al., 1988). During the last six years (2012–2018), 26 cases of pathogenic mutations in CaM have been reported, and all three *CALM* genes are now established major genes for both CPVT and LQTS (Table 1). All identified pathogenic CaM mutations cluster in the C-lobe, except the CPVT-causing variant N54I. Interestingly, this is the mutation with the mildest effect on biophysical parameters of CaM, including Ca^2+^ binding affinity. It is also the only variant found in a large family. Further, all LQTS-causing CaM mutations strongly reduce the C-lobe Ca^2+^ affinity, and, except for the F142L mutation, are all located in Ca^2+^-coordinating residues. This suggests that CaM mutations that strongly affect C-lobe Ca^2+^ affinity lead to LQTS (Table 1).

To date, there are more published cases of pathogenic mutations in *CALM1* (11) and *CALM2* (11) compared to *CALM3* (4). This could either reflect that the first published mutations were found in *CALM1* and *CALM2* and thus these two genes were included in genetic screening panels before *CALM3.* Or, it may be due to some subtle functional differences between the three genes. Quantitative PCR have shown that the three genes are not expressed at equal levels in cardiomyocytes, but the relative levels are not clear: whereas one study found higher levels of *CALM3* transcripts in human hearts (Crotti et al., 2013), the *CALM1* transcript was the most abundant in human stem cell-derived cardiomyocytes (Rocchetti et al., 2017).

As the number of published pathogenic CaM mutations has increased, several conclusions about genotype–phenotype relationships begin to form. Of particular interest is the CaM-D130G mutation, which has been identified in four unrelated individuals; two carrying the mutation in *CALM1*, one in *CALM2*, and one in *CALM3*, and all four suffering from LQTS. Similarly, the CaM-F142L mutation was found in both *CALM1* and *CALM3*, and all carriers suffered from LQTS. These observations imply that the amino acid position and type of change is important for the phenotype, and not the genetic origin of the transcript (*CALM1, −2*, or −*3*). One intriguing observation does, however, challenge this simple genotype–phenotype conclusion. The CaM-N98S mutation was found in *CALM1* in one individual, and in three other individuals in *CALM2*. Interestingly, these four patients present with different phenotypes – either CPVT or LQTS or both – suggesting that we still do not fully understand the underlying mechanisms determining the disease phenotype. These may involve other genomic variants able to shape the phenotype, complex protein regulatory effects, or environmental factors.

Since the protein products from all *CALM* genes are identical, it is tempting to speculate if a deletion of one allele (equivalent to a loss-of-function mutation) is less pathogenic than missense mutations. This idea immediately poses a therapeutic solution to patients carrying a CaM missense mutation, for example using the CRISPR/Cas9 technology to delete the pathogenic allele. Two studies specifically silenced the mutated CaM allele in patient-derived pluripotent stem cells differentiated into cardiomyocytes. Here, the CaM-D130G and -N98S mutations were silenced in *CALM2* with a partial or almost full restoration of Ca_V_1.2 regulation (Limpitikul et al., 2017; Yamamoto et al., 2017). These experiments are proof-of-principle that removal of the diseased allele may be a therapeutic solution. Also, these studies suggest that potential frameshift mutations causing premature stop codons or protein degradation may not be as detrimental as missense mutations. It still needs to be determined, however, if individuals carrying a loss-of-function mutation in a *CALM* gene are in fact unaffected from disease. Interestingly, a large exome sequencing study [Exome Aggregate Consortium (ExAC)] (Lek et al., 2016), found that *CALM1* and *CALM2* are intolerant to loss-of-function mutations (pLI = 0.89 and 0.86 respectively).

## Digging Deeper May Reveal a Broader Impact

Given the ubiquitous role of CaM, it is striking that all mutations identified are associated with a strong cardiac phenotype. We speculate whether these cases were discovered because of their unusual severity and because cardiologists and geneticists have specifically screened for *CALM* mutations in populations with cardiac disorders. Looking for CaM mutations in other patient groups may reveal new aspects and consequences of these mutations.

In a database containing variants from a large sequencing effort of almost 140,000 individuals (GnomAD, Lek et al., 2016), additional rare CaM missense mutations are reported (Figures 1B,C, gray residues and circles). The number of coding variants for all three genes is much lower than expected by chance. However, the cumulative frequency of additional rare CaM mutations suggests that CaM variants do not exclusively cause severe cardiac arrhythmias. At present, there is no overlap between variants identified in GnomAD (database variants) and the published pathogenic mutations. Further, the GnomAD missense variants are distributed throughout the entire protein, and more evenly distributed on the three *CALM* genes (9, 9, and 12 mutations in *CALM1*, −*2*, and −*3*, respectively), compared to the arrhythmogenic CaM variants. Also, all GnomAD variants except two, fall outside Ca^2+^-coordinating residues. Taken together, we therefore speculate that some of these uncharacterized variants are associated with unknown traits not involving cardiac arrhythmia. Sequencing results from large cohorts with known phenotypes are required to confirm this hypothesis.

We propose that studies of tissues other than cardiac are warranted for future research on the effects of CaM mutations. In particular, CaM expression is high in excitable neuronal cells. Also Ca_V_1.2 is widely expressed in neuronal tissues. Here, Ca_V_1.2 plays a role in cellular firing as well as in gene regulation, and mutations in Ca_V_1.2 have been attributed to psychiatric diseases (Nyegaard et al., 2010; Nanou and Catterall, 2018). Neurons express a number of other Ca_V_ channel isoforms, including Ca_V_1.3 and Ca_V_2 variants, which are also regulated by CaM. RyR2 plays a less prominent role in neurons, where the inositol triphosphate receptor (IP_3_R), which is also regulated by CaM, is the dominating intracellular Ca^2+^ release channel. Mild neuronal defects have been observed in some patients with CaM mutations, but these effects were suggested to be secondary, resulting from the frequent and severe episodes of syncope or cardiac arrest (Crotti et al., 2013; Boczek et al., 2016; Pipilas et al., 2016).

## Conclusion and Outlook

Within the last six years, CaM mutations have emerged as a novel cause of human diseases, the calmodulinopathies. All described pathogenic mutations have been identified in patients suffering from severe arrhythmic disorders, and biochemical as well as cellular studies have demonstrated that particularly the regulation of the Ca^2+^ channels Ca_V_1.2 and RyR2 are affected by these mutations. Currently, there is a strong correlation between LQTS-causing CaM mutations and Cav1.2 dysregulation, whereas all mutations affect RyR2 function. Given the ubiquitous role of CaM in a vast number of cellular processes, we predict that yet other targets may be affected. Our database search revealed a number of uncharacterized CaM missense mutations with unknown phenotypic consequences present in the population. Future studies will reveal whether other protein targets as well as other disease phenotypes can be assigned to mutations in CaM.

## Author Contributions

HJ wrote the first draft of the manuscript. HJ, MB, MN, and MO contributed to the content and writing. HJ and MB prepared the figure and table. All authors have read and approved the manuscript.

## Conflict of Interest Statement

The authors declare that the research was conducted in the absence of any commercial or financial relationships that could be construed as a potential conflict of interest.

## Abbreviations

AP: action potential
CaM: calmodulin
Ca_V_: voltage-gated calcium channel
CDI: calcium-dependent inactivation
CPVT: catecholaminergic polymorphic ventricular tachycardia
IVF: idiopathic ventricular fibrillation
LQTS: long QT syndrome
RyR2: ryanodine receptor isoform 2
SCD: sudden cardiac death
SR: sarcoplasmic reticulum
